# HIV-2 Protease resistance defined in yeast cells

**DOI:** 10.1186/1742-4690-3-58

**Published:** 2006-09-06

**Authors:** Najoua Ben M'Barek, Gilles Audoly, Didier Raoult, Pablo Gluschankof

**Affiliations:** 1 Unité des Rickettsies, Faculté de Médecine, 27 bd Jean Moulin, 13385 Marseille cedex 05, "Pathologies Transmissibles et Pathologies Infectieuses Tropicales", IFR48, France

## Abstract

**Background:**

Inhibitors of the HIV-1 Protease currently used in therapeutic protocols, have been found to inhibit, although at higher concentrations, the HIV-2 encoded enzyme homologue. Similar to observations in HIV-1 infected individuals, therapeutic failure has also been observed for some patients infected with HIV-2 as a consequence of the emergence of viral strains resistant to the anti-retroviral molecules. In order to be able to define the specific mutations in the Protease that confer loss of susceptibility to Protease Inhibitors, we set up an experimental model system based in the expression of the viral protein in yeast.

**Results:**

Our results show that the HIV-2 Protease activity kills the yeast cell, and this process can be abolished by inhibiting the viral enzyme activity. Since this inhibition is dose dependent, IC_50 _values can be assessed for each anti-retroviral molecule tested. We then defined the susceptibility of HIV-2 Proteases to Protease Inhibitors by comparing the IC_50 _values of Proteases from 7 infected individuals to those of a sensitive wild type laboratory adapted strain.

**Conclusion:**

This functional assay allowed us to show for the first time that the L90M substitution, present in a primary HIV-2 isolate, modifies the HIV-2 Protease susceptibility to Saquinavir but not Lopinavir. Developing a strategy based on the proposed yeast expressing system will contribute to define amino acid substitutions conferring HIV-2 Protease resistance.

## Background

Human Immunodeficiency Virus Type 2 (HIV-2), the second causative agent of the acquired immunodeficiency syndrome (AIDS), is mainly present in West Africa, where it was discovered [1] and spread to Europe, Asia, and the Americas in a slow but continuous manner. Although the two types of HIV (1 and 2) share only 40% of their amino acid sequences, HIV-2 infected individuals in developed countries are treated with highly active anti retroviral therapy (HAART), following the same therapeutic protocols that have been defined for HIV-1 infection.

HAART targets two main viral enzyme activities, the Reverse Transcriptase and the Protease. The drugs inhibiting the Protease competitively bind the substrate binding site of the enzyme, thus abolishing the proteolytic maturation of the Gag and Gag-pol precursors, resulting in the production of immature, non infectious particles [2].

Many epidemiological studies on HIV-1 infected individuals have documented that following anti-retroviral treatments a number of resistant isolates emerge causing therapeutic failure [3]. Resistant HIV-1 Proteases present a specific pattern of amino acid substitutions that are categorized as major or minor mutations [4].

Thus far the analysis of the data obtained from the few studies of the impact of Protease Inhibitors (PI), on HIV-2 infected individuals is still not consistent enough to clearly define the specific amino acid substitutions conferring resistance to these anti retroviral drugs. Nevertheless, the few reported data establish that i) the natural nucleotide polymorphism of the HIV-2 Protease includes amino acid substitutions that are associated with drug resistance in HIV-1 [5], and ii) comparison between the Protease sequences of treated and untreated HIV-2 infected individuals reveals a number of mutations under some PI-selective pressure such as K7R, V20I/A, I36V, V46I, I54L/M, V62A/T, V71L, I82F, I84V/L, 90LM, and L99F [6-8]. In a recent report, where five primary HIV-2 isolates obtained from two different infected individuals at different time points were tested for PI activity, it has been shown that neither I36V, V46I nor V71L modified the susceptibility of HIV-2 Protease to PIs [9]. Results, obtained from functional test on the ability of those PI to inhibit viral replication showed that amino acid substitutions such as : T12P [10], G17Q [10], R72A [10], M76V [10], I54M [7,9], I82F [7-9], V47A [9], K45R [9] confer a resistant phenotype.

In this study we present a novel test to assess isolated Protease susceptibility to PIs. This assay is based on the expression of the viral Protease in yeast cells and the definition of IC_50 _values for the PIs. As a proof of principle for this assay, we report the susceptibility pattern to Lopinavir and Saquinavir of HIV-2 Proteases from 7 infected individuals.

## Results

With the aim of designing a tool capable of defining the susceptibility of any viral Protease from HIV-2 treated or untreated infected individuals to different PIs in a cellular context, we exploited the yeast cell. Indeed *Saccharomyces cerevisiae *has been used for many decades as an experimental tool to study the functional role of several bacterial and viral proteins through the phenotype of the resulting transformed cells. Moreover, it has recently been shown that the HIV-1 Protease expressed in yeast induces cell lysis by a yet unknown mechanism unrelated to apoptosis [11].

The proteases encoded by the two HIV types are not identical, neither in their amino acid sequence nor in the specificity of peptide bond recognition [12]. Furthermore since there is no information concerning the cellular molecular pathway involved in the HIV protease-specific lysis of yeast cells, we first tested the phenotype induced by the expression of the HIV-2 Protease in yeast transformed cells. For this purpose we sub-cloned the Protease gene of the ROD isolate of HIV-2 in the pRS316Gal1/10 vector [13] under the control of the galactose inducible Gal 1/10 promoter [14], as detailed in the Material and Methods section.

**Figure 1:**
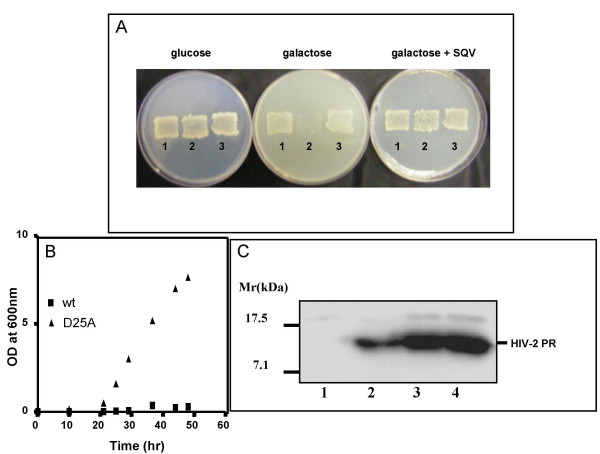
HIV-2 Protease expression in yeast induces cell growtharrest. A) BY4741 cells transformed either with pRS316Gal1/10 (1), pRS316Gal1/10-HIV2PR (2) or pRS316Gal1/10-HIV2PR-D25A were plated on minimal selective media containing glucose and replica-plated on galactose containing media in the presence or the absence of 200 μM Saquinavir. Yeast patches were observed after 2 days incubation at 30°C. B) 0.25 OD_600 _of yeast cells transformed either with HIV-2_ROD _Protease (wt), or with a genetically inactivated version of HIV-2_ROD _Protease (D25A), were incubated in 5 ml of liquid SGalC-Ura for 60 hours. At defined time points cell growth was measured (OD_600_). C) Soluble yeast cell extracts obtained from 1OD_600 _of growing cells were run on a SDS 17% PAGE, and subjected to Western blot analysis. 1: BY4741 [pRS316Gal1/10-HIV2PR] grown in glucose, 2: 25 ng of purified recombinant HIV-2 PR [34], 3: BY4741 [pRS316Gal1/10-HIV2PR(D25A)] grown in galactose, 4 : BY4741 [pRS316Gal1/10-HIV2PR] grown in galactose in the presence of 60 μM LPV.

HIV-2_ROD _Protease transformed yeast grew on glucose containing plates, but not on a galactose carbon source (Fig 1A). When an HIV specific protease inhibitor, such as Saquinavir (SQV) at 200 μM concentration, was added to the culture media (Fig 1A), the lethal phenotype was no longer observed on galactose. The galactose induced cell death was found to be directly linked to the protease enzyme activity, since when the Asp residue of the active site of the viral enzyme was modified to Ala (D25A mutant), the inactive Protease expressed in transformed cells (Fig 1C, lane 3) did not induce cell death when grown on galactose (Fig 1B). This implies that the protease activity was responsible for cell death. Additional evidence supporting that it is indeed this viral enzymatic activity which kills the yeast cells, and not simply the production of the exogenous protein, was provided through the analysis of the expression of HIV-2_ROD _Protease in transformed yeast which grew in galactose in the presence of 60 μM of the protease inhibitor Lopinavir (LPV). Western Blot analysis of cytoplasmic protein extracts from those cells showed an 11 Kd band recognized specifically by a monoclonal antibody raised against the viral Protease (Fig 1C, lane 2). As expected, this band was absent when the transformant was grown in glucose (Fig 1C, lane 1).

**Figure 2:**
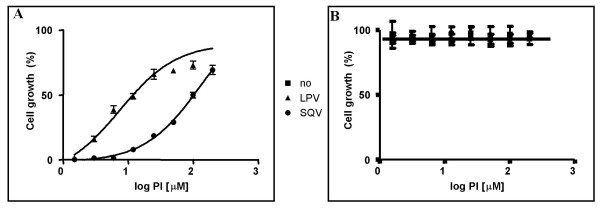
Susceptibility of HIV-2_ROD _Protease to Protease Inhibitors in transformed yeast 0.02 OD_600_/well of BY4741 [pRS316Gal1/10-HIV2PR] cells were either incubated in Galactose (A) or Glucose (B) containing synthetic media in the presence of increasing amounts of LPV and SQV (from 1.5 μM to 200 μM) in a 96 well plate. After 48 h at 30°C cellular growth was determined by measuring cell density at 600nm and plotted against PI concentrations. Results are presented as the percentage of cell growth; [(OD_600 _cells grew in Galactose with PI – OD_600 _cells grew in Galactose)/OD_600_cells grew in Glucose] × 100.

Based on this observation, we further studied the susceptibility of the Protease from the ROD isolate to LPV and SQV. Equal amounts of transformed cells were incubated in galactose, in the presence of increasing amounts of LPV or SQV. Cell growth was scored 48 h later by measuring the absorbance of the cell culture at 600nm. There was a tight correlation observed between the inhibitor concentration and cell growth which allowed determination of the corresponding IC_50 _values, for LPV 16.6+/-2.5 μM, and for SQV 149.7+/-7.2 μM (Fig 2A). These IC_50 _values were defined as the inhibitor concentration where cell growth reached 50% of regular growth in glucose. The growth arrest was not due to a toxic action of the PIs on the cells, since pRS316Gal1/10-HIV2PR transformed yeast incubated in glucose, in the presence or the absence of each PI (Fig 2B), or cells containing the pRS316Gal1/10 plasmid incubated in galactose, in the presence or the absence of PI (data not shown) grew identically.

As a first approach to validate this experimental system we measured the ability of LPV and SQV to inhibit HIV-2_ROD _Proteases harbouring resistant mutations. Out of a library of mutated HIV-2_ROD _Proteases that we created (see Material and Methods), we were able to select 3 mutants, containing at least one of the 8 amino acid substitutions which were previously defined on clinical samples through functional studies as conferring resistance [7-10]. The susceptibility to LPV and/or SQV of those mutants was defined by measuring cell growth of transformed cells in galactose, in the presence of either 150 μM of LPV or 1mM of SQV, that corresponds respectively to 9.0 and 6.7 fold of the IC_50 _value of the wild type Protease. In these culture conditions, cell growth of HIV-2_ROD _Protease transformed yeast was lower when compared to cell growth in the control media containing glucose. The percentage of cell growth was 91.5% in the presence of LPV, and 75% when incubated with SQV (Table 1). When this HIV-2_ROD _Protease harbours amino acid substitutions known to be involved in HIV-2 PI resistance such as K45R, I54M, or M76V [9,10], cell growth, in spite of the presence of the inhibitors, was strongly arrested achieving in some cases only 4% of normal growth (Table 1) demonstrating a tight correlation between resistance, defined in physiological conditions, and loss of susceptibility, defined in our experimental system.

**Table 1:** Protease Inhibitor resistance of HIV-2 Proteases tested in yeast

HIV-2_ROD _sequence	Phenotype (virus production)		Phenotype (transformed yeast) (% cell growth)	
	
	LPV	SQV	LPV	SQV
wild type	**S**	**S**	91.5+/-1.2	75.0+/-7.0
**K45R**	**R **[9]	**R **[9]	50.3+/-3.0	39.0+/-4.3
V20A, **I54M**	**R **[9]	**R **[9]	17.1+/-0.7	25.7+/-0.8
**M76V**	**n.d.**	**R **[10]	0.0+/- 2.4	4.0+/-0.2

The susceptibility of mutated HIV-2_ROD _Proteases, harbouring amino acid substitutions known to confer a resistant phenotype, were assessed in yeast in the presence of LPV at 150 μM and SQV at 1mM.

The resistant (R) or sensitive (S) phenotype is referred to previously published data.

Results in the yeast system are presented as the percentage of cell growth; [(OD_600 _cells grew in Galactose with PI – OD_600 _cells grew in Galactose)/OD_600 _cells grew in Glucose] × 100.

Using this assay, the susceptibility of HIV-2 Proteases from infected individuals was determined. We amplified the protease from DNA extracted from peripheral blood lymphocytes from 7 infected individuals either undergoing successful antiretroviral treatment or experiencing treatment failure (Table 2), as described in Material and Methods. For correct expression in yeast, an ATG start codon was introduced before the first Protease amino acid, and a TAA stop codon preceded by a Leu codon (as in HIV-2_ROD _Protease) was introduced after the 98^th ^codon. The DNA fragments obtained were sub cloned into the pRS316Gal1/10 expression vector, and the resulting plasmids were used to transform BY4741 yeast cells. Yeast transformants expressing HIV-2 Proteases were tested for their ability to grow in galactose in the presence of LPV and SQV (Table 3). We observed that at a concentration of 150 μM of LPV, one yeast strain presented cell growth similar to the ROD isolate (expressing MRT-29 viral Protease), three presented cell growth comparable to growth observed in glucose containing media (expressing MRT-7, -22, -25 viral Proteases), two (expressing MRT-8, -20 viral Proteases) showed a slightly lower growth than the ROD isolate, and only the yeast transformant expressing the MRT-1 Protease was impaired in its growth. When the same transformed cells were incubated in the presence of 1mM SQV, four of them showed the same phenotype as the ROD isolate (MRT-1, -20, -22, -29), two showed a cell growth comparable to the one obtained in glucose containing media (MRT-7, -25), and only yeast transformant containing the MRT-8 Protease grew to 55% compared to the control (Table 3). We further defined the IC_50 _values of the viral enzyme activity from those patients (Table 3). Interestingly, among the different samples analysed, only MRT-8 presented an IC_50 _to SQV 4.2 fold greater than the reference strain. Although definition of specific sensitive/resistant cut-off values (IC_50 _patient isolate/IC_50 _sensitive-strain) for each PI can only be obtained after a clinical study, the IC_50 _ratio we found for MRT-8 expressing Protease correlates to reported findings where loss of sensitivity corresponded to a higher IC_50 _value of about 4 fold, compared to the sensitive wild type isolate [15]. It should be remarked that :i) the HIV-2 Protease isolated from patient MRT-25 who never received SQV, showed a hypersensitivity towards this inhibitor (IC_50_MRT-25/IC_50_HIV-2_ROD _= 0.03), and ii) the Protease from patient MRT-1 showed a loss in sensitivity to LPV which might not be related to resistance since the IC_50 _ratio was found to be lower than 4.

**Table 2:** Protease Inhibitor treatment received by HIV-2 infected individuals

**Patient**	**Protease Inhibitor**	**CD4 (cells/μl)**	**Viral load (Log**_10_**)**	**Therapeutic failure**
MRT-1	NFV	173	3.9	yes
MRT-7	RTV, NFV	335	4.5	yes
MRT-8	SQV, IDV/rtv, NFV	211	5.3	yes
MRT-20	IDV, NFV	229	2	no
MRT-22	NFV, LPV	106	3.9	yes
MRT-25	IDV, LPV/rtv, APV/rtv	82	3.9	yes
MRT-29	NFV	53	4.5	yes

The Protease Inhibitors sequentially received by each patient from a previous cohort [6] are listed. (For this study, blood samples were taken on April 2002 for MRT-1, March 2002 for MRT-7, June 2003 for MRT-8, December 2000 for MRT-20, June 2002 for MRT-22, February 2003 for MRT-25, and May 2002 for MRT-29). Therapeutic failure was defined when the CD4 counts were < 200 and/or the viral load remained unchanged after treatment [6].

**Table 3:** Definition of HIV-2 Protease susceptibility to LPV and SQV, from infected individuals

	Phenotype (transformed yeast) (% cell growth)		IC_50 _[μM]		IC_50_isolate/IC_50_ROD	
Protease	LPV	SQV	LPV	SQV	LPV	SQV
ROD	91.5+/-1.2	75.0+/-7.0	16.6+/-2.5	149.7+/- 7.2	1.00	1.00
MRT-1	50.0+/-3.0	71.0+/-5	48.0+/-3.2	200.1+/-5.1	2.89	1.34
MRT-7	100.0+/-4.7	98.0+/-6	10.8+/-5.0	68.9+/-6.2	0.72	0.46
MRT-8	82.0+/-7.5	**55.0+/-6.3**	12.5+/-1.5	553.9+/-4.2	0.66	**4.23**
MRT-20	71.5+/-1.2	78.0+/-3.0	23.2 +/-4.4	118.0+/-6.3	1.40	0.79
MRT-22	98.5+/-1.0	82.0+/-7.1	15.1+/-4.3	104.8+/-7.1	0.91	0.70
MRT-25	100.0+/-2.0	**100.0+/-9.4**	19.9+/-4.6	48.5+/-4.7	1.20	**0.03**
MRT-29	93.0+/-2.0	72.0+/-3.0	13.9+/-5.5	153.1+/-3.0	0.84	1.02

Viral Protease genes from HIV-2 infected individuals, as well as from the ROD isolate, were PCR amplified, sub-cloned in pRS316Gal1/10 expression vector and used to transform yeast cells. Protease susceptibility to LPV and SQV was determined by defining the % of cell growth in galactose in the presence of inhibitors (as in table 1), and IC_50 _values were defined as in Fig 2A.

**Figure 3:**
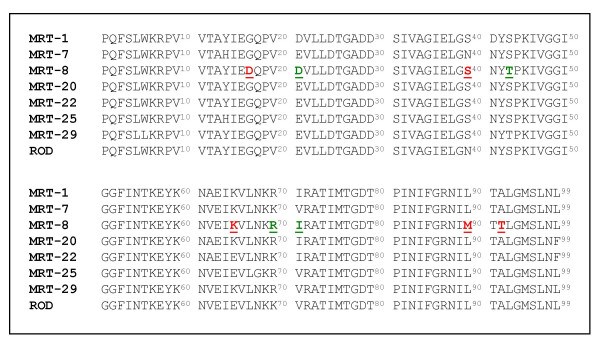
Amino acid sequences of Proteases from HIV-2 infecting isolates. PCR amplified HIV-2 Protease coding regions from 7 infected individuals were sequenced as described in Material and Methods and translated to amino acid sequence. Conservatives amino acid substitutions are in green while non-conservatives are in red.

In an attempt to define the amino acid mutation/s specifically responsible/s for the SQV loss of sensitivity phenotype of the MRT-8 Protease, we sequenced the coding DNAs of the 7 different Proteases and compared the sequences obtained to that of HIV-2_ROD _Protease (Fig. 3). Among the 9 mutations observed in the MRT-8 Protease (4 conservative and 5 non-conservative), we focused on the 90^th ^residue since the L90M substitution has already been classified as a major mutation in HIV-1 which confers resistance to SQV and LPV [4], and has been suggested to be associated with resistance to a yet undefined PI in HIV-2 Protease [8,16]. The HIV-2_ROD _Protease was mutated and the resultant ROD L90M mutant was tested for its susceptibility to LPV and SQV in yeast as was performed for HIV-2 Proteases from infected individuals. At the same time we created and tested the ROD L99F mutant, since this mutation was proposed to confer PI resistance in a study that scored the emergence of mutations in infected individuals failing an anti-protease containing regime [6], and no functional data of this suggested resistance is available. The results obtained, presented in Table 4, show that the L90M mutant have lost sensitivity to SQV but not to LPV (IC_50_HIV-2_ROD _L90M/IC_50_HIV-2_ROD_wt = 3.7 and 0.75 respectively). Conversely, the Phe residue in position 99 did not modify the respondent character of the Protease to either of the tested PIs (IC_50_HIV-2_ROD _L99F/IC_50_HIV-2_ROD_wt = 0.67 for LPV and 0.98 for SQV).

**Table 4:** Definition of mutated HIV-2_ROD_Protease susceptibility to LPV and SQV

	IC_50 _[μM]		IC_50_mutant/IC_50_ROD	
HIV-2 Protease mutant	LPV	SQV	LPV	SQV
L90M	12.5+/-8.1	554.1+/-3.9	0.75	**3.70**
L99F	11.1+/-3.1	147.2+/-5.1	0.67	0.98

L90M and L99F HIV-2_ROD _mutated Proteases were expressed in yeast. IC_50 _values to LPV and SQV were defined as in Fig 2A.

Through the data presented we have showed that the loss of HIV-2 Protease sensitivity to LPV and SQV can easily be evaluated by defining the IC_50_on protease-expressing yeast cells. Developing similar strategies to measure the IC_50 _values for other PIs in yeast constitutes the next step necessary to clearly define the overall resistance phenotype of any HIV-2 Protease.

## Discussion

The causal relationship between the increase of the viral load in plasma and the emergence of HIV isolates that are resistant to protease inhibitors and other anti retroviral compounds has been well established for HIV-1 infected individuals experiencing therapeutic failure [8,17,18]. In relation to HIV-2 infection, there is still a lack of information concerning the amino acid substitutions that confer resistance to various PIs. Current phenotyping methods for defining the sensitivity of an isolate to PIs, are founded on the production of a virus that contains the PCR amplified gene encoding the Protease isolated from the infected individual in a wild type genetic backbone [reviewed in 19]. This chimeric virus is then tested for its infectivity in the presence of PIs, and the IC_50 _values are obtained. This technology is complex and requires a significant infrastructure which is not well adapted for screening an increasing number of HIV-2 Proteases from infected individuals. As a way to define the biochemical resistance phenotype of viral proteases, we set up a simple and accurate experimental yeast cellular system to evaluate Protease sensitivity to antiretroviral compounds.

Our initial observation in this study was that the expression of the Protease encoded by HIV-2 in yeast cells induces cell death, as previously shown for the HIV-1 homologue [11]. This is not the only example of viral proteases that arrest cell growth in *S. cerevisiae*. Previous reports show that the 2A proteases from poliovirus and human rhinovirus 2 [20,21], both species belonging to the *Picornavidae *genus, produce cell growth arrest leading to cell death 10 h after their expression in yeast. These studies did not elucidate the specific protease-induced molecular cascades involved in cell death, but suggest the inhibition of RNA synthesis but not of translation as a key mechanism [20,21]. Although HIV-1 Protease possesses unique structural and functional properties that distinguish it from its cellular aspartic counterparts [22], several cellular proteins have been found to be efficient substrates. Among those, are proteins of the intermediary filaments, cytoskeleton components such as vimentin, desmin and glyal fibrillary acidic protein or cytoskeletal proteins as actin, troponin, tropomyosin [23,24], and microtubule-associated proteins [25], or bcl-2 [26], and precursors of NF-κB [27]. Human cells expressing the HIV-1 Protease die via apoptosis [26], however the lethal effect of this enzyme activity in yeast is likely to involve other death pathways since there is no evidence of apoptosis in *S.cerevisiae *[28]. It can thus be hypothesized that *S. cerevisiae *cell lysis produced by the viral Protease might occur through a drastic modification and/or degradation of the cellular cytoskeleton, as it does in higher eukaryote cells.

The mechanism by which HIV Proteases induce cell death in yeast is still an issue to be clarified, however since inhibition of Protease enzyme activity restores cell growth in HIV-2 Protease transformed yeast cells, we were able to define the IC_50 _values of the Protease from HIV-2_ROD _to LPV and SQV: 16.6+/-2.5 μM and 149.7+/-7.2 μM respectively. The difference between these values and those already reported in the literature, 27 nM and 11 nM respectively [9,29], might come from the nature of the yeast cell architecture. Indeed, the plasma membrane of the yeast cell has a non-human lipid composition that accounts for a different cell permeability. Moreover, the nature of the yeast cell wall might be a barrier for the entry of molecules that are hardly soluble in aqueous solutions, as the PIs.

The novel experimental system we present in this study allowed us to define the biochemical resistance phenotype to LPV and SQV for isolates from HIV-2 infected individuals either experiencing therapy failure or not. Moreover for the first time we were able to clearly define that the L90M substitution is responsible for SQV resistance but does not alter LPV sensitivity.

Our study also points out the importance of defining a PI susceptibility phenotype, as opposed to relying on the genotype, for definition of PI resistance/sensitivity in HIV-2 Protease isolates. The L99F substitution has previously been proposed to be involved in PI resistance, based on comparison of HIV-2 Protease sequences from PI untreated and treated patients [6]. However, in our phenotyping system, the L99F substitution did not modify susceptibility either to LPV or to SQV. This, highlights the limitations of comparative genotyping procedure in determining susceptibility of HIV-2 Proteases to PI and in predicting the ability of a given PI to inhibit isolates containing particular mutations. Establishing PI susceptibility phenotypes is the most accurate method to determine whether an HIV-2 virus strain is sensitive to a specific inhibitor used in a therapeutic protocol.

Our work is a proof-of-concept setting up and evaluating a phenotypic test to define protease susceptibility to PIs for HIV-2. The next step will include clinical validation to establish correlations with clinical outcomes and evaluate the predictive value for therapeutic failure.

## Materials and methods

### Patients

Patients were selected from a previous cohort of HIV-2 infected individuals being treatedat different hospitals in Marseilles and the surrounding area [6]. CD4 cell counts and viral load determination of each sample were assessed [6].

### Nucleic acid extraction and purification

Whole blood was collected in tubes containing EDTA. Peripheral blood mononuclear cells (PBMC) were separated from blood samples collected in EDTA by Ficoll-Hypaque centrifugation (Eurobio, Les Ullis, France). Aliquots containing 1×10^6 ^to 5×10^6 ^PBMC measured by cell count were frozen as dry pellets at -80°C until they were processed. The PBMC pellets were thawed, and total DNA was extracted using a QIAamp DNA minikit (Qiagen). Prepared DNA was directly analyzed or stored at -80°C.

### Construction of mutated HIV-2 Protease library

Mutated HIV-2 Proteases were generated by PCR using the Diversify PCR Random Mutagenesis Kit (BD Biosciences Clontech) on HIV-2_ROD _DNA. The amplification reaction was performed following the manufacturer instructions with primers BN-3 and NS. The obtained PCR products were cloned as a mutated library in pRS316Ga-RH plasmid through yeast transformation in a one step procedure. pRS316Ga-RH is a NotI linearisation product of pRS316Ga-RH-HIV2. pRS316Ga-RH-HIV2 is a modification of pRS316-Gal1/10 plasmid, where the BamHI-SacI polylinker region was replaced by the HIV-2_ROD _Protease flanked in its 5' extremity by the sequence of oligonucleotide BN-3, and in its 3' end by the sequence of the oligonucleotide SN.

BN-3 contains a BamHI and NotI restriction sites, underlined, and a start codon, in bold : CGAGGATCCGGAGACACCATACAGGGAGCCACCAACAGCGGCCGCGCC**ATG**CCTCAATTC. NS contains a SacI and NotI restriction sites, underlined, and a stop codon, in bold : GCGGAGCTCGCTTTAGCATTATTTTTATTGGCTCTACTGCGGCCGC**TTA**TAGATT.

Subcloning of the PCR products in the expressing vector was done as follows, in a one step procedure taking advantage of the homologous recombination event in yeast. Co-transformation of linear pRS316Ga-RH plasmid and the PCR mutated library was performed. Transformants living in glucose but dying in galactose in the presence of either 150 μM LPV or 1mM SQV were selected and the harboured viral Proteases sequenced.

### Viral DNA PCR amplification

Protease and RT genomic regions were amplified in a 50-μl reaction mixture under the conditions recommended by the manufacturer. Nested PCR was performed using 1 to 10 μl of purified DNA, 50 pmol of inner primers (forward: H2Mp3, 5'-ACTTACTGCACCTCGAGCA, 2,020 bp; reverse: H2Mp4, 5'-CCCAAATGACTAGTGCTTCTT, 3,527 bp), to obtain a genomic fragment of 1,507 bp. The PCR products were analyzed in a 1.5% agarose gel with ethidium bromide.

### HIV-2 Proteases

The DNA fragments coding for the different HIV-2 proteases were amplified by PCR, either from HIV-2_ROD _clone14 [30] or from the PCR amplified 1507 bp fragment from HIV-2 infected PBMC extracted DNA [6]. The primers used were : Fwd, CAGAGGATCCGCT**ATG**CCTCAATTCTC, that contains a BamHI site followed by a start (bold underlined) codon 5' to the first amino acid of the Protease sequence, and Rev, CCGGAC**TTA**TAGATTTAATGACATGCC, that contains a stop codon 3' to the last protease encoded Leu amino acid. The 412 bp length product was blunt ended, purified by WIZARD PCR Preps DNA purification kit (PROMEGA), subcloned in pGEM T easy vector, and further sub-cloned in pRS316GAL1/10 expression vector [12] as a BamHI-SacI fragment.

The genetically inactivated HIV-2_ROD _Protease (D25A) was constructed by 3 consecutive PCR reactions on HIV-2_ROD _clone14. The first PCR, produced a DNA fragment coding for a truncated protease starting at its 14^th ^amino acid and carrying the D25A mutation. The second PCR added to the resulted DNA fragment the sequence coding for the amino acids 8^th ^to 13^th^. The last PCR added the sequence coding for the amino acids 1^st ^to 7^th^, preceded by a start codon and 5' flanked by a BamHI site. The final amplified DNA fragment was sub-cloned into pRS316GAL1/10 expression vector following the same procedure as described for the wt gene. The 3' primer used in all three reactions was Rev. The different 5' primers were: for the first PCR : F14-28, ACATTGAGGGTCAGCCAGTAGAAGTTTTGTTA**GCC**ACGGGAGC, where the GAC codon, coding for Asp in position 25, was changed to GCC (bold underlined) coding for Ala. For the second PCR: F8-17, AAGACCAGTAGTCACAGCATACATTGAGGG. For the third PCR: FB1-9, CAGAGGATCCGCT**ATG**CCTCAATTCTCTCTTTGGAAAAGACCAG.

All PCR products were purified using the WIZARD PCR Preps DNA purification kit (PROMEGA).

The L90M mutant was constructed by PCR using the primers Fwd and 3-90LM. 3-90LM, CCGGACTTATAGATTTAATGACATGCCTAAGGCTGT**CAT**AATATTTCTGCC, contains the Met codon at position 90 (bold underlined) and a stop codon 3' to the last Protease encoded amino acid. The obtained product was purified and sub-cloned as a BamHI-SacI fragment in pRS316GAL1/10.

The L99F HIV-2 Protease was constructed by PCR using the primers Fwd and pL99F. pL99F, a reverse primer, CCGGACTTA**GAA**ATTTAATGACATGCC, contains a Phe codon (bold underlined) at position 99 of the HIV-2 Protease, followed by a stop codon. The product was purified and sub-cloned as a BamHI-SacI fragment in pRS316GAL1/10 expression vector following the same procedure as for the wild type Protease.

### Yeast transformation

Yeast strain BY4741 (*MATa, hisΔ 1, leu2Δ 0, met15Δ 0, ura3Δ 0*) obtained from EUROSCARF [31] was transformed following the Lithium Acetate procedure [32].

### Inhibition of HIV-2 Protease and IC_50 _determination

BY4741 yeast cells harbouring the DNA encoding the different HIV-2 Proteases were grown overnight at 30°C in liquid SDC-URA medium to exponential phase. 2.5 OD_600nm _were harvested and washed twice with sterile water and re-suspended in 25 ml of SGalC-URA (supplemented minimal yeast nitrogen base with galactose instead of glucose). 0.02 OD_600nm _were seeded in each well of a 96 micro-well plate in the presence or absence of a Protease Inhibitor. The plates were incubated 48h at 30°C and cell growth was estimated by measuring the optical density at 600 nm with a TECAN Genesis RSP100 (Tecan Inc., Research Triangle Park, N.C). Cell growth, as % of cells growing in glucose containing media = [(OD_600nm _of cells grown in Galacatose and PI containing media – OD_600nm _of cells grown in Galacatose)/OD_600nm _of cells grown in Glucose] × 100, were plotted against Protease Inhibitor concentration and the IC_50 _value was defined as the inhibitor concentration that rescues 50% of cell growth. Experiments were performed in triplicate.

### Protease inhibitors

The Protease Inhibitors used in this study are a kindly gift from Abbott Laboratories (for Lopinavir) and from Roche Diagnostics GmbH, Mannheim, Germany (for Saquinavir).

### Western blot analysis

10 ml of yeast cell grown to exponential phase were harvested, centrifuged and lysed using glass beads in Laemmli buffer as previously described [33]. Solubilized proteins were resolved on SDS 17% PAGE and transferred onto nitrocellulose membrane. Immunoblotting was carried on with a mouse monoclonal antibody recognizing HIV-1 and -2 proteases ab8327 (Abcam), followed by peroxydase-conjugated goat anti-mouse antibodies (Jackson ImmunoResearch). The HIV-2 protease was detected using the ECL kit (Amersham Biosciences, Upsala, Sweden).

### DNA sequencing

pRS316Gal1/10 vectors harbouring viral Proteases from infected individuals were used as DNA template for nucleotide sequence with primers GAL (TGCATAACCACTTTAACT), hybridising with the 5' upstream region to the viral gene, and M13F (GTTTTCCCAGTCACGACG) hybridising with the 3' downstream region to the viral gene. The protease gene was sequenced in both directions with the Big dye Terminator version 1.1 cycle sequencing Ready Reaction PCR kit (PerkinElmer, Coignières, France). Resulted products were purified on MultiScreen-PCR Filter Plate (Millipore, Saint-Quentin en Yvelines, France) and sequenced on an Applied Biosystem automatic sequencer model 3100 (PerkinElmer).

## Abbreviations

Lopinavir (LPV), Saquinavir (SQV), Protease Inhibitor (PI), Highly active anti retroviral therapy (HAART).

## Competing interests

The author(s) declare that they have no competing interests.

## Authors' contributions

NBM and GA performed the experiments. PG wrote the manuscript and participated with NBM and GA in the experimental design and data interpretation. DR provided the clinical specimens and participated in data interpretation. All authors read and approved the final manuscript.
